# Functional Changes during Hospital Stay in Older Patients Admitted to an Acute Care Ward: A Multicenter Observational Study

**DOI:** 10.1371/journal.pone.0096398

**Published:** 2014-05-12

**Authors:** Stefanie L. De Buyser, Mirko Petrovic, Youri E. Taes, Davide L. Vetrano, Andrea Corsonello, Stefano Volpato, Graziano Onder

**Affiliations:** 1 Department of Geriatrics, Ghent University Hospital, Ghent, Belgium; 2 Department of Endocrinology and Unit for Osteoporosis and Metabolic Bone Diseases, Ghent University Hospital, Ghent, Belgium; 3 Centro Medicina dell'Invecchiamento, Università Cattolica del Sacro Cuore, Rome, Italy; 4 Unit of Geriatric Pharmaco-epidemiology, IRCCS - Italian National Research Centre on Aging (INRCA), Cosenza, Italy; 5 Department of Medical Sciences, University of Ferrara, Ferrara, Italy; Bielefeld Evangelical Hospital, Germany

## Abstract

**Objectives:**

Changes in physical performance during hospital stay have rarely been evaluated. In this study, we examined functional changes during hospital stay by assessing both physical performance and activities of daily living. Additionally, we investigated characteristics of older patients associated with meaningful in-hospital improvement in physical performance.

**Methods:**

The CRiteria to assess appropriate Medication use among Elderly complex patients project recruited 1123 patients aged ≥65 years, consecutively admitted to geriatric or internal medicine acute care wards of seven Italian hospitals. We analyzed data from 639 participating participants with a Mini Mental State Examination score ≥18/30. Physical performance was assessed by walking speed and grip strength, and functional status by activities of daily living at hospital admission and at discharge. Meaningful improvement was defined as a measured change of at least 1 standard deviation. Multivariable logistic regression models predicting meaningful improvement, included age, gender, type of admission (through emergency room or elective), and physical performance at admission.

**Results:**

Mean age of the study participants was 79 years (range 65–98), 52% were female. Overall, mean walking speed and grip strength performance improved during hospital stay (walking speed improvement: 0.04±0.20 m/s, p<0.001; grip strength improvement: 0.43±5.66 kg, p = 0.001), no significant change was observed in activities of daily living. Patients with poor physical performance at admission had higher odds for in-hospital improvement.

**Conclusion:**

Overall, physical performance measurements show an improvement during hospital stay. The margin for meaningful functional improvement is larger in patients with poor physical function at admission. Nevertheless, most of these patients continue to have poor performance at discharge.

## Introduction

Functional changes in older persons with an acute illness can be expected during hospital stay [1], [2]. However, changes in physical performance during hospital stay have scarcely been evaluated. In the existing literature, in-hospital functional changes have been almost exclusively reported by changes in functional status [3]–[6], e.g. using the Barthel Index [7] and Katz's Activities of Daily Living (ADL) index [8]. Functional status measurements are self-reported and their accuracy can be affected by the complex circumstances of hospital stay. Sager *et al.* [9] found discrepancies between patient' assessments and performance-based measurements of the ability to do ADLs in a substantial proportion of hospitalised older persons. Bathing and dressing were the two activities in which agreement rates were the lowest. For example, patients who need help dressing because they are tethered to an intravenous pole, may have a clouded judgment about the ability to perform this ADL independently [5].

Alternatively, physical performance measurements (PPMs) can be used to assess physical function in older adults. PPMs can even identify more limitations in physical functioning than self-reported subjective measurements [10]. Furthermore, PPMs are more sensitive to change and might be more useful for longitudinal evaluations [11]. Finally, PPMs are more able to predict outcomes than self-reported measurements [12]. Results of functional change during hospital stay might be different for PPMs compared with ADL index [5], [9].

Past studies have mainly focused on functional decline of acutely ill older patients during hospital stay [13]–[15]. Functional decline is strongly associated with nursing home admission [16] and 3-month mortality [6]. Nevertheless, functional improvement after hospital admission has also been reported [4]–[1].

This study had two objectives. The first objective was to examine functional changes during hospital stay in older patients admitted to geriatric or internal medicine acute care wards by assessing both physical performance and functional status. The second objective was to investigate which characteristics of older patients are associated with meaningful in-hospital improvement in physical performance.

## Methods

### Ethics statement

The study complies with the ethical rules for human experimentation that are stated in the Declaration of Helsinki. All participating hospitals (Gemelli Hospital, Università Cattolica del Sacro Cuore in Rome/University of Perugia/University of Ferrara/Italian National Research Center on Aging (INRCA) in Ancona/INRCA in Cosenza/INRCA in Fermo/INRCA in Rome) had obtained approval for the study from their ethical committee. Written, informed consent was obtained from all participants.

### Data source & study population

Data from the CRiteria to assess appropriate Medication use among Elderly complex patients (CRIME) project were used. The CRIME project was initiated to assess prescribing patterns in older adults hospitalised across Italy and to produce recommendations for appropriate pharmacological prescribing in older complex patients. Details about the methodology of the CRIME project are reported elsewhere [17]–[19].

CRIME participants were patients aged 65 years or more, consecutively admitted to geriatric or internal medicine acute care wards of the seven above mentioned hospitals. Between June 2010 and May 2011, a total number of 1123 hospitalised older in-patients were enrolled in the CRIME project.

### Data collection

A questionnaire was designed to assess the participants within 24 hours of admission and at daily intervals until discharge. Study researchers had received a two-day training course in which they were well-trained about how to correctly collect and report questionnaire data. The study researchers used a variety of information sources, including direct observation, clinical records, and interviews with the patients, family, friends or formal service providers. The questionnaire included demographics, type of admission (through emergency room or elective if planned previously), anthropometrics, socio-economics, cognitive status (30 items Mini-Mental State Examination (MMSE) [20]), psychological status (15 items Geriatric Depression Scale), drug use, medical diagnoses, and geriatric conditions (pain, falls, delirium, and pressure sores). Data on drug use, medical diagnoses and geriatric conditions were updated daily.

### Physical function measurements

Physical function was assessed within the first 24 hours after hospital admission and the day of discharge by the study researchers. Walking speed (WS) was assessed by having the participant walk at his/her usual pace over a four-meter distance. This test has shown a high test-retest reliability [12]. For the present study the fastest walk of two measurements was used in the analyses. Not all patients were ambulatory at admission. For this reason WS assessment was not performed in 228 patients.

Measurement of grip strength (GS) was performed using a North Coast Medical hand dynamometer. Patients were seated with the wrist in a neutral position and the elbow flexed 90°. In case a subject was unable to sit, GS was assessed lying at 30° in bed with the elbows supported. The highest value of two consecutive measurements obtained with the dominant hand was used in the analyses. A distinction was made between subjects unable to perform GS and subjects who did not execute the test despite being capable.

Dependency in ADLs (transferring, bathing, dressing, eating, bowel and bladder continence, and personal hygiene) was reported to assess functional status just before admission. Scores ranged from no to six dependencies.

### Analytical approach

In order to exclude patients not able to complete the PPMs because of cognitive problems or inability to understand instructions, analyses were limited to patients with an MMSE score ≥18/30. This is in line with other projects focusing on physical performance in older persons [21]. Further, patients who died during hospital stay (N = 25) were excluded from the analyses. This left an analytical sample of 639 subjects. Various sub-analyses have been performed, for example, by excluding subjects unable to perform WS or GS at admission or by excluding subjects with high performance at admission (WS ≥0.8 m/s or GS ≥20 kg/30 kg for women/men).

Functional change was computed in the way that positive values indicate a functional improvement. In order to capture functional change in subjects unable to perform a test at admission or discharge, the value corresponding to the first percentile of admission performance of participants was assigned to these subjects and to those with a performance below the first percentile (WS: 0.23 m/s, GS: 5 kg). Subjects who did not perform GS despite being capable, were treated as missing variables (N = 4). Meaningful improvement in physical performance was defined as a measured change of at least 1 standard deviation (SD), this equals a 0.20 m/s increase in WS and 5 or 7 kg increase in GS for women or men, respectively. For functional status, change in the ability to do at least 1 ADL was considered meaningful.

To visualise the functional change according to admission performance, subjects were categorised into three groups according to physical performance at admission. WS categories were: unable to perform the test, less than 0.8 m/s, and at least 0.8 m/s [22]. GS categories were: unable to perform the test, less than 20 kg in women or 30 kg in men, and at least 20 kg in women or 30 kg in men [22].

### Statistical methodology

Continuous variables were expressed as mean ± SD or median (first to third quartile), where appropriate. Countable variables were presented as absolute number and percentage (%) of the study population. In-hospital change in physical function was examined with paired samples T-tests or related-samples Wilcoxon signed rank test, where appropriate. Multivariable logistic regression was used to predict meaningful improvement in WS and GS. Regression models included age, gender, type of admission, and physical performance at admission (continuous variable). Additional analyses also included length of stay (days), MMSE score, comorbidity (sum), or number of drugs during stay. Hosmer-Lemeshow goodness-of-fit tests indicated no signs of a bad model fit. All analyses were performed using SPSS software, version 19.0 (SPSS Inc., Chicago, IL). Differences according to type of admission in ability to perform PPMs and in physical performance of participants were examined with Chi-square statistics and Independent-Samples T tests, respectively. The relationship between comorbidity and physical performance at admission was assessed using linear regression analyses. Statistical significance was indicated by a P value <0.05; all P values were two-tailed.

## Results

### Patient Characteristics

Age ranged between 65 and 98 years. Men and women were nearly equally represented (48% men). Slightly more than half of the patients were electively admitted (55%). Detailed characteristics of our sample are reported in Table 1. Most prevalent diseases were hypertension (N = 523, 82%), ischemic heart disease (N = 206, 32%), heart failure (N = 156, 25%), diabetes mellitus (N = 195, 31%), osteoarthritis (N = 239, 37%), chronic obstructive pulmonary disease (N = 237, 37%), and renal failure (N = 147, 23%).

**Table 1:** Characteristics of the study population (N = 639).

	Value
Age (years), mean ± SD	79.2±6.9
Gender (female), N (%)	331 (52)
Elective admission, N (%)	349 (55)
Walking speed category at admission, N (%)	
Unable to perform the test	228 (36)
<0.8 m/s	291 (46)
≥0.8 m/s	120 (19)
Grip strength category at admission, N (%)	
Unable to perform the test	78 (12)
<20 kg ♀/<30 kg ♂	368 (58)
≥20 kg ♀/≥30 kg ♂	189 (30)
ADL dependencies, median (IQR)	1 (0–2)
Length of stay (days), median (IQR)	9 (6–14)
MMSE, median (IQR)	25 (22–28)
Geriatric Depression Scale (15-items), median (IQR)*	4 (2–7)
Comorbidity sum, median (IQR)	4 (3–6)
N° drugs during stay, median (IQR)	9 (7–13)

*Geriatric Depression Scale data were missing for 48 subjects.

SD =  standard deviation; ADL =  Activities of Daily Living; IQR =  interquartile range; MMSE =  Mini-Mental State Examination.

Within patients admitted from the emergency room, 49% (N = 141) and 22% (N = 53) was unable to perform WS or GS, respectively. These proportions are substantially higher (P<0.001) than those in patients admitted electively (N = 87, 33% and N = 25, 8%). In patients able to perform, mean GS and WS performance did not significantly differ according to type of admission.

### Changes during Hospital Stay in Physical Performance and Functional Status

Overall, mean WS and GS performance improved significantly during hospital stay (Table 2), but most patients had no meaningful change in WS (86%, N = 552) or GS (88%, N = 558). Thirty-six% of subjects (N = 228) were unable to perform WS at admission, of these 23% (N = 52) regained their ability with a mean WS of 0.69±0.28 m/s at discharge. Twelve% was unable to perform GS at admission, of these 41% (N = 78) had regained function at discharge (mean GS = 17.48±10.61 kg). The mean and SD of GS change was larger in men than in women (1.02±6.65 kg vs. −0.12±4.50 kg). Table 2 provides more details concerning the changes in physical performance during hospital stay. Sub-analyses excluding subjects unable to perform WS or GS at admission led to similar mean changes.

**Table 2:** In-hospital change in physical performance.

		Admission Scores		In-hospital Change				
	N	mean	SD	mean	SD	% change vs. admission SD a	P	meaningful improvement % (N) b
**Walking speed (m/s)**	639	0.52	0.29	0.04	0.20	13.79	<0.001	10 (62)
**Grip strength (kg)**	635	19.16	10.36	0.43	5.66	4.15	0.001	7 (45)

a Percent change vs. standard deviation (SD) of the mean was calculated with the following formula: 100*mean change/SD of mean at admission.

b Meaningful improvement was defined as ≥0.20 m/s walking speed and ≥5 kg ♀/≥7 kg ♂ grip strength.

SD =  standard deviation.

Globally, functional status, expressed by ADL score, did not significantly change during hospital stay, only 38 subjects improved in ADL (P = 0.058). The great majority of the subjects (91%, N = 581) obtained the same score of admission at discharge (median admission score = 1 (0–2) dependencies).

### Characteristics Associated with Meaningful Changes during Hospital Stay

**Figure 1:**
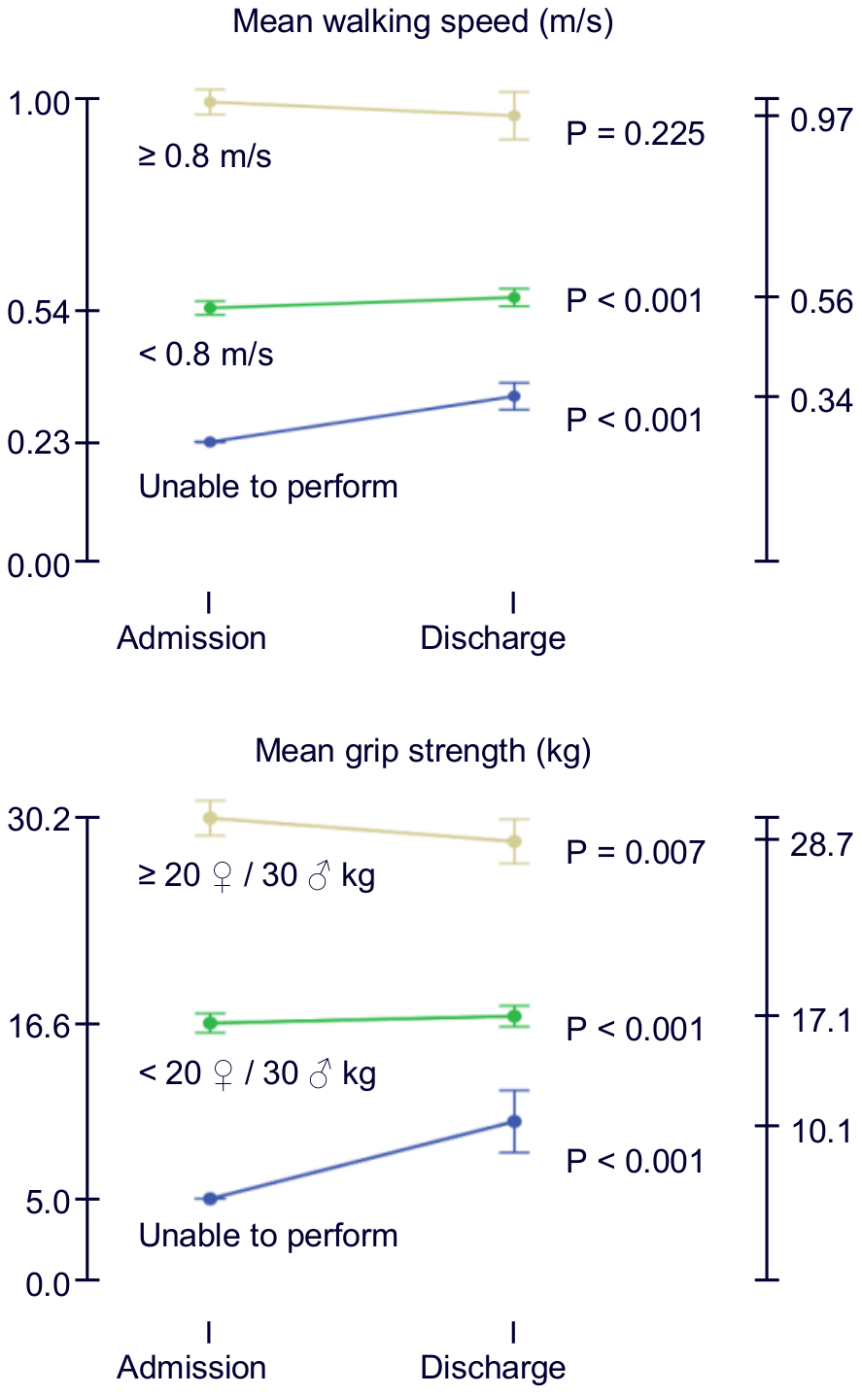
Change in physical performance measurements according to admission performance. Error bars represent 95% confidence intervals.

As illustrated in Figure 1, in-hospital change in physical function varied according to admission performance. Subjects who were unable to perform WS at admission or with slow WS (<0.8 m/s) improved in mean WS performance during stay, but remained to have poor function at discharge. Similarly, subjects who were unable to perform GS or with weak GS at admission improved in mean GS performance during stay, but still performed poorly at discharge. Subjects with high GS performance at admission had a significant decline during hospital stay.

Table 3 shows the results from multivariable logistic regression analyses predicting meaningful improvement in WS or GS during hospital stay. These models illustrate an association of admission performance with functional improvement during hospital stay. The odds for in-hospital improvement decreased when patients were electively admitted and when they had higher performance at admission. Additionally, the odds for WS improvement during stay decreased with older age. Sub-analyses excluding subjects with high performance at admission led to similar results. Additional analyses including one extra covariate found length of stay (days), MMSE score, and number of drugs during stay not to be predictive of functional improvement. Higher comorbidity was associated with higher odds for meaningful improvement in GS (OR = 1.02, CI_95_ = 1.04–1.33, P = 0.009). Comorbidity sum was also significantly associated with GS performance at admission (β = −0.51, CI_95_ = −0.84–−0.19, P = 0.002).

**Table 3:** Associations with meaningful improvement in physical performance during hospital stay.

	Walking speed improvement			Grip strength improvement		
	OR	CI_95_	P	OR	CI_95_	P
**Age (years)**	0.95	0.92–0.99	0.022	0.98	0.93–1.02	0.320
**Gender (male)**	1.48	0.87–2.55	0.148	1.70	0.89–3.26	0.110
**Elective admission**	0.42	0.24–0.74	0.003	0.46	0.23–0.93	0.030
**Admission performance (m/s or kg)**	0.19	0.06–0.57	0.003	0.86	0.82–0.91	<0.001

Data reported are from multivariable logistic regression models predicting improvement of ≥0.20 m/s in walking speed and improvement of ≥5 kg ♀/≥7 kg ♂ in grip strength.

OR =  odds ratio; CI_95_ = 95% confidence interval.

## Discussion

The *first* objective of this study was to examine functional changes during hospital stay in older patients admitted to acute care. Because functional change has scarcely been evaluated by PPMs, we have assessed both WS and GS performance at admission and at discharge.

PPMs have mostly been used in community-dwelling older persons, where a WS of 0.8 m/s has been accepted to define low WS [22]. Ostir *et al.* [23] assessed WS in acutely ill older patients admitted to acute care. They found 64% of patients could complete the WS test, with a mean performance of 0.53±0.25 m/s [23]. Their results are in perfect agreement with ours. Common gender-specific thresholds for GS to identify mobility limitations are 20 kg/30 kg [22] and 21 kg/37 kg [24] for women/men.

Due to older inpatients' acute illness, high catabolism, bed rest, sleep deprivation, and polypharmacy, hospital stay is a risk factor for functional decline [1], [2]. Nevertheless, we found an overall mean improvement in physical performance during hospital stay, while median functional status (ADL score) did not change significantly.

The detected improvement in physical performance might be part of a functional recovery trajectory, where functional improvement is preceded by functional decline before hospital admission as a consequence of the acute medical illness [4], [5]. Stabilisation of the acute medical condition may outweigh the negative consequences of hospital stay on physical function [25].

The overall improvement in physical performance, observed in our study, is in line with the results of Volpato *et al*. [11] who reported in-hospital change in performance on the Short Physical Performance Battery [26] of 92 patients; 63% had better performance at hospital discharge. Similarly, Bodilsen *et al.* [25] reported an improvement during hospital stay in mean physical performance of 33 patients, quantified by the Timed Up and Go test. Furthermore, Purser *et al.* [27] reported a mean improvement in WS during stay of 0.03 m/s in frail older veterans.

Unlike two others studies [25], [28], we could validate a significant improvement in mean GS performance during hospital stay. These other studies either excluded subjects unable to perform PPMs [25] or assessed changes after only one week of hospital stay [28]. In our study, subjects unable to perform had the greatest functional change and 65% of subjects stayed in hospital longer than seven days.

The *second* objective of this study was to investigate which patient characteristics are associated with meaningful in-hospital improvement. In our study, improvement in physical performance was related to admission performance, with poor performers experiencing meaningful improvements more frequently than good performers. These poor performers might have had a functional recovery trajectory with greater functional decline before admission. Since, in the study of Palleschi *et al*. [4], greater functional decline before hospital stay was a significant predictor of in-hospital functional improvement. In addition, a floor effect may clarify the observed improvement in poor performers, given that subjects unable to perform at admission could not further decline. When interpreting these results, one must consider that regression toward the mean might be responsible for improvement in poor performers and decline in good performers.

Older subjects had lower odds for WS improvement. Similarly, in the study of Covinsky *et al.* [5], older patients were more likely to fail to recover in ADL function during hospital stay.

Subjects who were electively admitted had lower odds to improve performance. Patients admitted from the emergency room often present with severe acute conditions, which may have led to a steep decline in physical performance before hospital admission. During hospital stay they can recover from the acute conditions and consequently improve their level of physical performance during stay. Patients admitted electively are less likely to present severe acute conditions. Therefore, they are less likely to improve during stay. Similarly, subjects with higher comorbidity might have a larger margin to recover from an acute condition than those with few diseases.

### Performance-based versus patient-reported physical function

As reported in other studies, performance-based and patient-reported measurements of physical function appear to assess distinct and only partially overlapping domains of physical function [29], [30]. Diehr *et al.* [31] found WS to be the most sensitive indicator of age-related decline in older adults. In our study, changes in WS and GS could be detected over the short period of time in hospital. On the contrary, the 6-item ADL scale did not seem suitable to assess in-hospital changes. Our results suggest that PPMs might be more sensitive to demonstrate functional changes during hospital stay, than self-reported functional status. Use of PPMs in the acute care setting should be encouraged, as PPMs may provide important clinical information in acutely ill older subjects. Multifaceted aspects of aging are integrated in physical functions measurements, including disease processes, nutritional status, and fitness [12]. In addition, low physical performance may reflect a state of frailty.

### Limitations & Strengths

Our results have implications for the feasibility of PPMs in the acute care setting. We assigned a continuous value equivalent to the worst percentile of performance, to those patients who were unable to perform WS and GS. Just like Purser *et al.* [27], we found this to be a feasible way of tracking continuous improvement over time. Although this recoding may have introduced bias, we found that excluding those unable to perform led to similar mean changes. Unfortunately, the reason why subjects were unable to perform was not recorded. However, the exclusion of subjects with an MMSE below 18 removed patients not able to complete the test because of cognitive problems or inability to understand instructions. Therefore physical problems were the main reason why subjects were unable to perform PPMs.

An important variable not recorded is main reason of admission. The severity of the disease that led to hospital admission might very well be a confounding factor. However, we believe this factor is partially captured in the type of admission. Patients admitted from the emergency room often present with severe acute conditions, while patients admitted electively are less likely to present severe acute conditions. The high proportion of subjects unable to perform within patients admitted from the emergency room endorses this theory. Unfortunately, we do not have the data to fully explore these findings.

The percentage of subjects with meaningful change was relatively low. Our definitions of meaningful change (0.20 m/s WS and 5 or 7 kg GS women/men) seem roughly in line with those reported elsewhere [32]–[34]. Substantial meaningful change in 4-m WS observed in community-dwelling older adults was estimated at 0.10 m/s [32], while substantial meaningful improvement in WS observed during recovery from hip fracture was estimated between 0.17 to 0.26 m/s [33]. Estimates of meaningful change in WS may differ based on the direction of change or between patient populations [33]. Regarding GS, a change of more than 6 kg was suggested as necessary to detect a genuine change in GS 95% of the time [34]. It is conceivable that patients with relatively high performance on admission could not be able to demonstrate such meaningful improvement during hospital stay due to ceiling effects in PPMs. Sub-analyses have confirmed that predictive factors for functional change did not alter when subjects with high performance at admission were excluded.

Our study was restricted to functional changes from admission until discharge. Given the possibility that patients are admitted in the night, a 24-hour window was allowed to perform the first assessment. Medical therapy could have taken place between admission and assessment that could affect patients' physical performance. After hospital discharge, functional changes might still occur as part of the functional recovery trajectory. Volpato *et al.* [35] reported an improvement in 50% of patients in performance on the Short Physical Performance Battery [26] during the first month after discharge.

A strength of this study is the availability of comprehensive data. We present objective data in the clinical setting where PPMs have received little attention [35]. Our data demonstrate the feasibility of PPMs in acute care setting. Furthermore, we provide a better understanding of the dynamic nature of physical performance in older people with an acute illness during hospital stay. The multicentre design of the study improves generalisability of our results to acute care settings across Italy and Europe.

### Further research

Both in community-dwelling and hospitalised older subjects, physical function measurements have shown their predictive value in terms of various adverse health-related outcomes, such as mortality, institutionalisation, and healthcare costs [36]–[38]. Our results suggest that the interpretation of physical performance at a single time point is not straightforward. More research is needed to determine how functional changes can add value to the prediction of hospital outcomes. Functional trajectories might even be more prognostic than single and static measurements of physical function [6].

## Conclusions

This study was one of the few that observed in-hospital change in physical performance of older subjects. Overall, PPMs show an improvement during hospital stay. The margin for meaningful functional improvement is larger in patients with poor physical performance at admission. Nevertheless most of these patients continue to have poor performance at discharge.
